# Supplementary material for: Making Biomedical Research Software FAIR: Actionable Step-by-step Guidelines with a User-support Tool
Source: Sci Data. 2023 Aug 23;10:557. doi: 10.1038/s41597-023-02463-x (PMC10447492; doi:10.1038/s41597-023-02463-x)
Supplement: Supplementary file 1 — Supplementary Tables [file 41597_2023_2463_MOESM1_ESM.pdf]

**Supplementary Table 1**. The five categories we established based on the instructions derived for each of the FAIR4RS principles v1.0. The combined instructions for each category, the FAIR4RS principles they cover, and the outstanding questions for fulfilling these instructions are also provided.

| Category | Instructions | Questions for fulfilling the instructions |
|---|---|---|
| Category 1: Develop software following standards and best practices | Develop the software such that it meets domain-relevant community standards and coding practices, e.g., choice of programming language, standards for testing, usage of file format, accessibility by as many people as possible (R3). Develop the software such that the data it reads, writes, and exchanges (e.g., with other software) meets relevant community standards (e.g., data should be in a file format that is standard for that data type) (I1). | What are the relevant standards to follow when developing biomedical research software?<br><br>What are the relevant standards for data that the software reads, writes, and exchanges?<br><br>What are relevant coding best practices for developing biomedical research software? |
| Category 2: Include metadata | Include metadata that follows community standards and uses controlled vocabulary (F2). The metadata needs to include a plurality of attributes, i.e. use multiple terms for the same, similar, or overlapping concept (R1). The metadata is required to include several elements: relation between different versions of a software (F1.2), the identifier of the software and describes how it can be obtained (F3), information about citing the software (F4), the standards followed by the data interacting with the software (I1), qualified references to other objects required to run the software (I2), detailed provenance of the software (i.e., why and how the software came to be, as well as who contributed what, when and where) (R1.2), and qualified references to other software required to run the software (R2). Metadata needs to be included in both machine-readable and human-readable e.g. software documentation format (F4, R1). The documentation needs to meet domain-relevant community standards (R3). Version control systems such as GitHub can be used to record details of the software development history (R1). | What relevant standards exist for metadata format and structure (outside of repositories and registries) that allow to document the mandatory metadata in human and machine-readable format?<br><br>How can biomedical research software be documented following relevant standards (documentation format, documentation content)? |
| Category 3: Provide a license | Provide a clear license that is, preferably, widely used and as unrestrictive as possible (R1.1). The license must be provided such that it is readable by both humans and machines (R1.1), is compatible with the dependencies of the software (R1.1), and meets relevant standards (R3). | How to provide a license in human-readable and machine-readable formats that are standard? What are widely used licenses that are suggested? |

| | | |
|---|---|---|
| Category 4: Share software in a repository | Share software on a suitable repository that issues a unique and long-lasting identifier (F1), helps with including rich metadata that follow community standards and uses controlled vocabulary (F2), includes the identifier of the software, and describes how it can be obtained (F3) and is FAIR, searchable, and indexable (F4). A suitable repository can also help with making the software accessible via its identifier through a standardized protocol (A1) that is open and free (A1.1.) and allow for authentication and authorization when necessary (A1.2). Share such that different components (software, commits, files, etc.) of the software (F1.1) and different versions of the software (F1.2) are assigned distinct identifiers as deemed suitable by the developers. | What repositories can be used for archiving biomedical research software? In what format should the research software be archived? |
| Category 5: Register in a registry | Register the software on a suitable registry to make the software metadata accessible even when the software is no longer available (A2). A suitable registry can also act as an alternative or complement to a repository for obtaining a unique and long-lasting identifier (F1), including rich metadata that follow community standards and uses controlled vocabulary (F2), includes the identifier of the software and describes how it can be obtained (F3), and is FAIR, searchable, and indexable (F4). | What registries can be used for registering biomedical research software? |

**Supplementary Table 2**. Crosswalk table that explains how the FAIR-BioRS guidelines version 2.0.0 allow complying with the FAIR4RS principles v1.0.

| FAIR4RS Principles | Compliance through the FAIR-BioRS guidelines |
|---|---|
| F1. Software is assigned a globally unique and persistent identifier. | Archiving the software on Zenodo/Figshare (step 5.2) will assign a Digital Object Identifier (DOI) which is a unique and persistent identifier. Archiving the software on Software Heritage (step 5.3) will assign a SoftWare Heritage persistent IDentifier (SWHID) which is also a unique and persistent identifier. Bio.tools/RRID Portal will issue a unique and persistent identifier as well (bio.tools ID and RRID, respectively) when the software is registered (step 6). |
| F1.1. Components of the software representing levels of granularity are assigned distinct identifiers. | Bio.tools/RRID Portal (step 6) will assign a unique identifier for the entire software. Archiving each version of the software on Zenodo/Figshare (step 5.2) will assign a distinct identifier (DOI) for each version. Archiving the software on Software Heritage (step 5.3) will assign a distinct identifier (SWHID) to any level of granularity of the software (software, releases, files, commits, code fragments, etc.). |
| F1.2. Different versions of the software are assigned distinct identifiers. | Archiving each version of the software on Zenodo/Figshare (step 5.2) will assign a distinct identifier (DOI) for each version. Archiving on Software Heritage (step 5.3) will assign a distinct identifier for each version release of the software as well. Changes between versions will be documented in the CHANGELOG file (step 3.2). |
| F2. Software is described with rich metadata. | Rich metadata covering a variety of aspects will be provided through the code-level documentation (step 2.1), the dependencies recording (step 2.2), the instructed documentation (step 3), the prescribed metadata files (step 4), the repository-specific metadata on Zenodo/Figshare (step 5.2), and the registry-specific metadata on bio.tools/RRID Portal (step 6). |
| F3. Metadata clearly and explicitly include the identifier of the software they describe. | The README file will include the DOI from Zenodo/Figshare in a “How to cite” or similar section (step 3.1). The codemeta.json and CITATION.cff files (step 4) will include the DOI from Zenodo/Figshare in their "identifier" and "identifiers" fields, respectively. The DOI from Zenodo/Figshare is always included in that repository’s metadata (step 5.2). The DOI will also be included in the bio.tools/RRID portal’s metadata which also includes their respective IDs (step 6). |
| F4. Metadata are FAIR, searchable and indexable. | FAIR, searchable, and indexable metadata that follow community standards and use controlled vocabularies are provided through Zenodo (aligns with DataCite's Metadata Schema minimum and recommended terms, with a few additional enrichments)/Figshare (aligns with DataCite's Metadata Schema) (step 5.2), Software Heritage (follows the CodeMeta vocabulary) (step 5.3), and Bio.tools (uses the biotoolsSchema and EDAM ontology)/RRID Portal (follows the Resource Description Framework (RDF) and aligns with the Biomedical Resource Ontology (BRO) and the Eagle-i Resource Ontology (ERO) along with few additions) (step 6). The prescribed license documentation (step 1.2), development best practices (steps 2.1 and 2.2), documentation of the software (step 3), and prescribed metadata files (step 4) will contain additional metadata that also follow community standards, use controlled vocabularies, and is typically searchable through the suggested version system control platforms (step 1.1). |
| A1. Software is retrievable by its identifier using a standardised communications protocol. | The software archive can be retrieved by the DOI generated by Zenodo/Figshare (step 5.2) using HTTP, which is a standardized protocol. The software will be retrievable through the version control system platform (step 1.1), the deployment repository if applicable (step 5.1), and Software Heritage (Step 5.3) also using HTTP. |
| A1.1. The protocol is open, free, and | The HTTP protocol is open, free, and universally implementable. |

| | |
|---|---|
| universally implementable. | |
| A1.2. The protocol allows for an authentication and authorization procedure, where necessary. | Version control systems platforms (step 1.1), deployment repositories (step 5.1), and Zenodo/Figshare (step 5.2) have a process in place to allow for an authentication and authorization procedure for software shared under closed/restricted access. Everything on Software Heritage (Step 5.3) is open access and does not require any authentication or authorization. |
| A2. Metadata are accessible, even when the software is no longer available. | Once archived on Zenodo or Figshare (step 5.2) and on Software Heritage (step 5.3) both the software and metadata will always be available and accessible for the lifetime of these repositories. Moreover, Zenodo and Figshare send metadata from the software to DataCite for generating a DOI and that metadata will always remain accessible through DataCite's registry. Additionally, Zenodo keeps metadata stored in high-availability database servers separate from the software files. Bio.tools/RRID Portal (step 6) will also keep the metadata accessible even if the software is no longer available e.g., on the version control system platform or any of the archiving repositories. |
| I1. Software reads, writes and exchanges data in a way that meets domain-relevant community standards. | Step 2.4 will ensure that the inputs/outputs of the software follow any applicable community standards. Those standards will be documented in the README file under a “Standards followed” or similar section (step 3.1). They can also be documented in the bio.tools metadata using the EDAM ontology to specify the nature and format of the input and output data. |
| I2. Software includes qualified references to other objects. | The README file/documentation will contain qualified references to other objects associated with the software under a “Parameters and data required to run the software” or similar section (step 3.1). The fields “isPartOf”, “hasPart”, and “relatedLink“ of the codemeta.json file (step 4.1) will also provide qualified references to other objects. The Zenodo metadata (step 5.2) include a “Related identifiers” field that can be used to provide qualified references to other objects. |
| R1. Software is described with a plurality of accurate and relevant attributes. | The software will be described with a plurality of accurate and relevant attributes through the development history captured by the version control system platform (step 1.1.), the prescribed documentation (step 3), the prescribed metadata files (step 4), the repository-specific metadata (step 5), and the registry-specific metadata (step 6), which all have several overlapping elements. |
| R1.1. Software is given a clear and accessible license. | The software will be given a clear and accessible license through step 1.2 which instructs selecting a license and including a LICENSE file with usage terms. The metadata of the software repository in the version control system platform (step 1.1), the metadata files (step 4), the repository-specific metadata (step 5), and the registry-specific metadata (step 6) will all include the name of the license. |
| R1.2. Software is associated with detailed provenance. | Detailed provenance (why and how the software came to be, as well as who contributed what, when and where, etc.) will be provided in several ways: in the development history maintained by the version control system platform (step 1.1.) that will also get archived in Software Heritage (step 5.3), in the README through an “Overall description of the software” and a “How to cite” or similar sections (step 3.1), in the codemeta.json file through several fields such as Software description/abstract (“description”) and Authors (“givenName”, “familyName”) with their Organization name (“affiliation”) (step 4.1), in the CITATION.cff file through several fields such as Authors ("given-names", “family-names") with their Organization name (“affiliation”) (step 4.2), in the repository-specific metadata (step 5), and the registry-specific metadata (step 6). |
| R2. Software includes qualified references to other software. | The software dependencies file (step 2.2) will contain qualified references to other software required to run the source code. Following language-specific best practices (step 2.3) will also allow including dependencies in the code (e.g., imports in Python code). The README files (step 3.1) will contain qualified references to other software under a “High-level dependencies of the software” or similar section. The fields |

| | |
|---|---|
| | “isPartOf”, “hasPart”, and “relatedLink“ of the codemeta.json file (step 4.1) will provide qualified references to other software. The Zenodo metadata (Step 5.2) also includes a “Related identifiers” field that can be used to provide qualified references to other software. The bio.tools metadata (step 6) include a “Relations” class that can be used to provide qualified references to other software registered on bio.tools. |
| R3. Software meets domain-relevant community standards. | Steps 1, 2, and 3 will ensure that the software, including its documentation and license, meet domain-relevant community standards and best practices. Sharing software on a deployment repository (if applicable) will also help meet domain-relevant community standards and best practices (step 5.1). |
